# LiveDrive AI: A Pilot Study of a Machine Learning-Powered Diagnostic System for Real-Time, Non-Invasive Detection of Mild Cognitive Impairment

**DOI:** 10.3390/bioengineering12010086

**Published:** 2025-01-17

**Authors:** Firas Al-Hindawi, Peter Serhan, Yonas E. Geda, Francis Tsow, Teresa Wu, Erica Forzani

**Affiliations:** 1 School of Computing and Augmented Intelligence, Arizona State University, Tempe, AZ 85281, USA; falhinda@asu.edu; 2 ASU Mayo Center for Innovative Imaging, Arizona State University, Tempe, AZ 85281, USA; 3 School of Electrical, Computer and Energy Engineering, Tempe, AZ 85281, USA; pserhan@asu.edu; 4 Center for Bioelectronics and Biosensors, Biodesign Institute, Arizona State University, 1001 S McAllister Ave, Tempe, AZ 85281, USA; 5 Barrow Neurological Institute, 2910 N 3rd Ave, Phoenix, AZ 85013, USA; yonas.geda@commonspirit.org; 6 TF Health Corporation (DBA Breezing Co.), 6161 E. Mayo Blvd., Phoenix, AZ 85054, USA; frant@breezing.co; 7 School of Engineering for Matter, Transport and Energy, Arizona State University, Tempe, AZ 85281, USA

**Keywords:** Alzheimer’s disease, mild cognitive impairment, smart driving, machine learning

## Abstract

Alzheimer’s disease (AD) represents a significant global health issue, affecting over 55 million individuals worldwide, with a progressive impact on cognitive and functional abilities. Early detection, particularly of mild cognitive impairment (MCI) as an indicator of potential AD onset, is crucial yet challenging, given the limitations of current diagnostic biomarkers and the need for non-invasive, accessible tools. This study aims to address these gaps by exploring driving performance as a novel, non-invasive biomarker for MCI detection. Using the LiveDrive AI system, equipped with multimodal sensing (MMS) technology and a driving performance assessment strategy, the proposed work analyzes the predictive capacity of driving patterns in indicating cognitive decline. Machine learning models, trained on an expert-annotated in-house dataset, were employed to detect MCI status from driving performance. Key findings demonstrate the feasibility of using nuanced driving features, such as velocity and acceleration during turning, as indicators of cognitive decline. This approach holds promise for integration into smartphone or car applications, enabling real-time, continuous cognitive health monitoring. The implications of this work suggest a transformative step towards scalable, real-world solutions for early AD diagnosis, with the potential to improve patient outcomes and disease management.

## 1. Introduction

Alzheimer’s disease (AD), a distinct form of dementia, stands as one of the most widespread health challenges associated with aging, currently impacting over 55 million individuals globally [1,2]. AD is primarily recognized by its hallmark symptoms of memory loss, cognitive deterioration, and behavioral changes. As the disease progresses, these symptoms intensify, eventually leading to a profound impairment of an individual’s cognitive abilities, severely disrupting daily functioning and mental capacity [3]. This neurodegenerative condition is marked by a prolonged preclinical phase that can extend up to two decades before any noticeable symptoms emerge. During this asymptomatic stage, significant neuronal damage quietly unfolds, setting the stage for the cognitive decline that characterizes the later stages of the disease [4]. The early symptomatic stage of AD is characterized by cognitive decline, often identified as mild cognitive impairment (MCI) when clinicians attribute the decline to the prodromal stage of Alzheimer’s rather than other potential causes like different forms of dementia, medication effects, or depression [5]. MCI represents a transitional phase between normal aging and dementia, characterized by memory loss and cognitive difficulties that are more pronounced than what is typical for one’s age yet not severe enough to impact daily activities significantly; however, not all individuals with MCI will eventually develop AD [6]. While MCI is not a definitive diagnosis of AD, it is a critical diagnostic category as it serves as a strong predictor of future Alzheimer’s, with approximately 15% of MCI patients progressing to full-blown AD each year [7]. While no cure currently exists for AD, early diagnosis is crucial as it allows for the initiation of therapies that can slow the disease’s progression and enhance its overall management [8], as treatment strategies and medications are generally more effective during the initial stages of AD [9]. Given that there is currently no known cure for Alzheimer’s, taking early action is essential to slowing the disease’s progression and improving patient outcomes [10]. 

Diagnosing MCI presents a challenge, as cognitive decline is also a common feature of healthy aging. This challenge underscores the need for reliable diagnostic biomarkers for both MCI and AD. Among the most widely utilized measures to aid clinical decision-making is the assessment of medial temporal atrophy (MTA) through visual inspection of structural magnetic resonance imaging (MRI) scans [11,12]. However, MTA is not particularly useful for early diagnosis of MCI, as it typically becomes apparent only in the later stages of disease progression [13]. Other biomarkers, such as fluorodeoxyglucose or amyloid positron emission tomography (PET) and cerebrospinal fluid analysis, offer valuable insights but are often less accessible, more expensive, and more invasive compared to MRI [11]. Moreover, traditional cognitive assessments are typically infrequent and limited to a single point in time, which fails to capture the dynamic and multifaceted nature of cognitive functions, which are typically influenced by numerous factors. For example, stress experienced in daily life significantly impacts cognitive abilities [14]. Likewise, sleep patterns profoundly affect cognitive health, highlighting the intricate relationship between rest and cognitive processes [15]. Medications are also crucial in evaluating cognitive performance, as they can exert both direct and indirect effects on cognitive function [16]. Additionally, environmental stressors, ranging from pollution to social and emotional stress, play a pivotal role in shaping cognitive abilities, with the potential to alter cognitive function significantly [17,18]. Considering the limitations of existing diagnostic biomarkers, there is an urgent need to develop tools that can detect the early manifestations of cognitive decline and provide accurate diagnoses for both MCI and early AD in free-living conditions. Such an approach will provide a more detailed and accurate understanding of the progression of cognitive diseases, enabling earlier detection and more effective management of the condition.

Recently, researchers [19,20,21] have begun investigating the potential of using driving performance as a biomarker for AD. Driving is an inherently complex task that demands extensive neurocognitive engagement, requiring the seamless integration of various cognitive domains, including attention, memory, executive function, and visuospatial skills, all within a dynamic and ever-changing environment [22]. This complexity makes driving a practical and non-invasive method for assessing brain function and cognitive challenges, positioning it as a promising indicator for detecting early signs of cognitive impairment and neurodegenerative diseases.

With the rise in machine learning across diverse applications [23,24,25], its use in detecting MCI and AD is no exception. Di et al. [20] introduced a method for analyzing driving data using machine learning algorithms. This study collected data from 2977 participants through in-vehicle devices and utilized the Random Forest algorithm for classification. This study showcased significant predictive ability, achieving an F1 score of 0.88 by incorporating both demographic and driving data, indicating that, indeed, machine learning models driven by driving data present a promising method for the early detection of MCI and dementia. In a different study, Bayat et al. [19] utilized machine learning and GPS driving data to detect early signs of cognitive decline. The study conducted reported an F1 score of 0.82 when predicting preclinical AD using a Random Forest classifier trained solely on GPS driving data. The performance improved to 0.88 when age was also included in training the model. Roe et al. [21] conducted a study that spanned over 2.5 years with 20 participants to investigate the relationship between driving performance and preclinical AD in older adults. The study showed a correlation between preclinical AD and a general decline in driving activities such as miles covered, driving frequency, and the number of locations visited. These studies confirm that, indeed, preclinical AD manifests itself in driving activities, indicating the possibility of using it as a biomarker for MCI and AD diagnoses.

Although these studies reported successful results, they suffer from drawbacks. A significant limitation arises from the methodology used to treat monthly driving records as independent data points. This approach raises important concerns about the validity of the results from a machine learning modeling point of view as it introduces a risk of data leakage, allowing for the possibility that data from the same driver could be present in both the training and testing datasets. Even though the records may come from different months, the underlying driving behavior patterns unique to each individual could be learned by the model during training and inadvertently used to make predictions in the test set. This compromises the model’s ability to generalize to new drivers, potentially overestimating the reported performance metrics. Moreover, the features used by these studies are very simplistic and do not capture the true complexity of the driving task adequately. They focus on high-level driving behavior, such as trip frequency and trip duration, while not including behaviors that are more directly engaging with the mental capability of the subject, such as turn velocity, acceleration, frequency, and path deviation.

To address these issues, a more robust approach would involve ensuring that all data from a given driver are allocated exclusively to either the training or test set. This method preserves the model’s ability to generalize to unseen individuals, providing a more accurate assessment of its true predictive power. Additionally, it is essential to incorporate more driving-specific features that adequately capture the true complexity of the driving task. In response to these challenges, this work presents a pilot study on LiveDrive AI, an integrated system that leverages multimodal sensing (MMS) technology and is equipped with a suite of sensors, including an accelerometer, gyroscope, and GPS. Moreover, a driving performance assessment strategy is introduced to assess the subjects’ driving patterns during multiple driving maneuvers and under different stress level conditions, such as normal vs. aggressive driving. The pilot system is designed to objectively evaluate a wide range of driving behaviors and performance metrics across various decision-making levels. Unlike previous machine learning works that overlooked inter-subject variability, this approach accounts for inter-subject variability when training the models, which is a more difficult task to solve from a machine learning perspective. The system was trained and tested using an in-house dataset annotated with the collaboration of field medical experts. Different machine-learning models were used for comparison. This work uses individual-level driving data to develop a classifier to predict the risk of MCI for each study participant. The algorithm based on driving performance features could be incorporated into a smartphone app, car app, or other devices for early detection of MCI and dementia in older adult drivers in real time.

This pilot study lays the groundwork for future research aimed at transitioning from a controlled driving environment to a naturalistic, free-roaming setup, where participants’ daily driving performances can be observed in real-world conditions. A key objective of this phase is to identify specific driving performance features indicative of MCI, which will guide subsequent research and model development. In later phases, data from these naturalistic driving scenarios will be collected and analyzed to train a machine-learning model capable of detecting MCI in everyday contexts. Ultimately, this progression aims to achieve the final goal of integrating the LiveDrive AI system into a day-to-day application, enabling continuous and non-invasive cognitive health monitoring in individuals’ daily lives.

In summary, the key contributions of this manuscript are as follows:

- Driving Performance Data as a New Digital Biomarker: The system leverages MMS technology to capture test subjects’ driving performances in a manner that more effectively reflects the complexity of human brain engagement with real-world tasks.
- Standardized Driving Performance Assessment Strategy: A novel testing framework evaluates driving performance across multiple maneuvers and different stress levels (e.g., normal vs. aggressive driving) to gauge performance under varied conditions.
- Accounting for Inter-Subject Variability: The approach uniquely considers inter-subject variability, an often-overlooked critical factor in machine learning for cognitive decline, to avoid reporting inflated results.
- Pilot Testing for Real-Time Practical Application Potential: The model demonstrates its feasibility for integration into mobile applications and in-car systems, providing a practical, cost-effective, and non-invasive solution for the early detection of mild cognitive impairment (MCI) and dementia in older drivers.

This work is presented as follows: Section 2 outlines the Materials and Methods used in developing the LiveDrive AI system. Section 3 presents the results and discusses the key findings. Section 4 offers concluding remarks and proposes directions for future research. Finally, Section 5 lists the references that informed this work.

## 2. Materials and Methods

**Figure 1:**
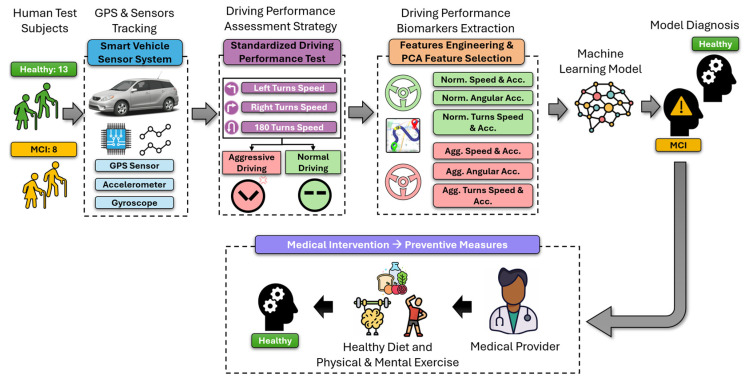
An overview of the proposed LiveDrive AI system: Once human subjects are diagnosed by a medical expert, they proceed through the system, which combines GPS and sensor tracking with a driving performance assessment strategy involving driving tests. This system analyzes the driving patterns of healthy and MCI subjects. Features such as speed and acceleration (Acc.) are extracted under normal (Norm.) and aggressive (Agg.) conditions from turning maneuvers, processed through machine-learning models for diagnosis, and linked to preventive medical interventions, including a healthy diet and physical and mental exercises.

The LiveDrive AI system is summarized in Figure 1. The system is designed with an array of hardware components to capture extensive driving performance data vital for assessing cognitive decline through driving performance. Central to the system are the accelerometer, gyroscope, and GPS sensors. These sensors are incorporated into a smartphone and a specialized BitBrew Inc.’s (Danlaw Inc., 41131 Vincenti Ct, Novi, MI, USA) Onboard Diagnostics (OBD) data logger. These tools capture dynamic driving data and performance parameters crucial for the assessment and allow data transmission in real time to a customized iOS application. The integration of these hardware components within the system and the performance driving maneuvers test allows for holistic monitoring of driving performance conditions, thereby enhancing the system’s ability to evaluate factors that may influence cognitive decline. This approach underlines the system’s effectiveness in monitoring and potentially mitigating factors contributing to cognitive decline in drivers. Each individual sub-component of the system will be discussed in detail in the following sub-sections.

### 2.1. Human Test Subjects

The study was designed to monitor and evaluate driving performance among older adults aged 65 to 85 years. Its primary goal was to identify early signs of cognitive decline, particularly MCI. The study involved twenty-one volunteers who were thoroughly educated about the study’s design and purpose. Each participant provided informed consent, following approval by the Institutional Review Board at Arizona State University (IRB protocol number: STUDY00006547). Table 1 provides a summary of the demographics and medical status of the participants in this study.

To ensure accuracy in the study, human subject annotations were made in collaboration with a neurologist with a specialty in AD. The volunteers were categorized into two distinct groups based on their cognitive health: one group of 13 participants was identified as cognitively healthy, while the other group of 8 participants had been diagnosed with MCI. This classification enabled a clear comparison between the driving performances of healthy individuals and those experiencing early cognitive decline, providing critical data for the system’s analysis.

### 2.2. Smart Vehicle Sensor System

**Figure 2:**
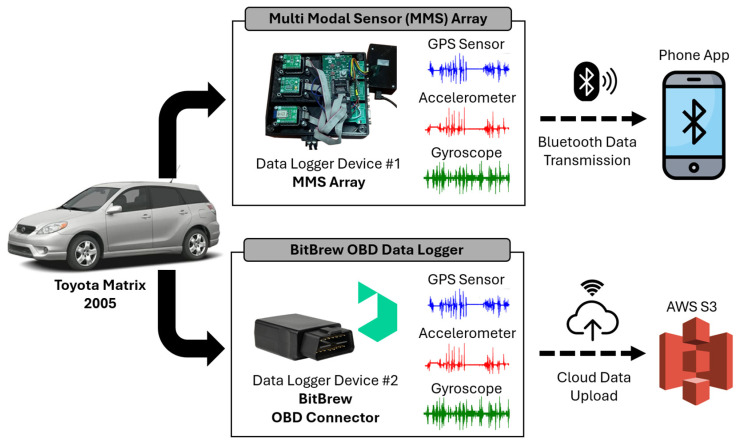
The smart vehicle sensor system setup: The test vehicle, a 2005 Toyota Matrix, was equipped with two backup data logging systems: an MMS array (Data Logger Device #1) and an OBD-connected device provided by BitBrew (Data Logger Device #2). Both loggers recorded data from three key sensors: a GPS sensor (1 Hz) for speed tracking, an accelerometer (10 Hz), and a gyroscope (10 Hz). Data from the MMS array were transmitted via Bluetooth to a smartphone within the vehicle, while the BitBrew device uploaded its readings directly to AWS S3 cloud storage.

The vehicle sensor system, depicted in Figure 2, employs a multimodal sensor array integrated into the vehicle to capture a wide range of driving performance data. This array consists of several critical sensors, including a GPS sensor, which tracks the vehicle’s location, speed, and route. Additionally, an accelerometer is used to measure the vehicle’s acceleration and deceleration patterns, providing insight into how the vehicle behaves during various driving conditions. Finally, a gyroscope complements this by capturing the vehicle’s angular movements, such as turns and rotations. The sampling rate for both the accelerometer and the gyroscope is 10 Hz, while the sampling rate for the GPS sensor is 1 Hz.

To facilitate real-time data assessment, the system is connected to a mobile application on a mobile device, which receives signals from the sensor array via Bluetooth. As a backup and to enhance data redundancy, the system is equipped with an onboard diagnostics (OBD) data logger from BitBrew Inc., which includes additional sensors (accelerometer, gyroscope, and GPS). These data are then transmitted to the cloud through Amazon Web Services (AWSs) and collected on the BitBrew platform, serving as an experimental alternative method for data collection. The entire sensor system is installed in a Toyota Matrix 2005. The printed circuit board (PCB) that houses these components is powered by the car’s 12 V battery, which is stepped down to 5 V to ensure the proper functioning of the sensors and other electronic components.

### 2.3. Standardized Driving Performance Assessment Strategy and Driving Performance Parameters Extraction

**Figure 3:**
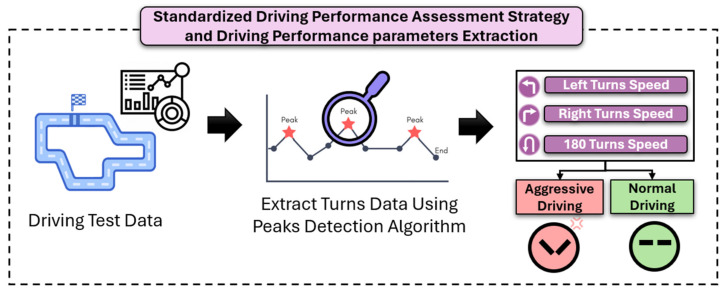
An overview of the standardized driving performance assessment strategy and driving performance parameters extraction is presented, detailing the process of generating driving data, applying a peak detection algorithm to isolate data specifically related to turning maneuvers, and extracting aggressive versus normal driving data based on instructions given to the test subjects during the standardized driving test experiment.

As shown in Figure 3, the system was designed to assess driving performance under two conditions: normal driving and aggressive driving. This study focused on critical actions like left turns, right turns, and 180-degree turns due to the significant cognitive demands these maneuvers impose. These actions were selected because they require complex mental processes, including spatial orientation, decision-making, and divided attention (abilities that are often affected early in AD). Successfully executing turns demands that drivers simultaneously monitor their environment, make real-time judgments, and adjust motor responses accordingly. These tasks strain cognitive domains such as working memory, spatial awareness, and processing speed, which are crucial for safe driving and are also commonly compromised in individuals with AD. By examining driving performance specifically during turns, this study aims to highlight subtle yet impactful cognitive impairments that may serve as early biomarkers for MCI, aiding in early detection and intervention. During these maneuvers, the MMS array closely monitored the driving performance to capture detailed data. The testing protocol involved evaluating driving performance under two distinct conditions: aggressive driving and normal driving. Aggressive driving was characterized by sharp turns, rapid acceleration, and hard braking, whereas normal driving was defined by smooth, controlled maneuvers that reflected typical, everyday driving performance.

Each participant was required to follow a standardized test on a predefined road circuit located at our Health Futures Center’s facility. The circuit was designed to simulate a variety of real-world driving scenarios, consisting of straight sections and a combination of 90-degree (right and left) and 180-degree turns. Each subject completed 10 laps of the circuit, with each lap measuring 540 m, ensuring a comprehensive evaluation of their driving capabilities under different conditions.

After the standardized test is completed, the recorded data undergo a comprehensive processing phase. The raw sensor data comprise high-precision measurements such as angular velocity (ω, in rad/s), acceleration (m/s^2^), GPS speed (m/s), and location coordinates. A key aspect of our methodology is analyzing the Z-axis angular velocity data from the gyroscope, as they are crucial for detecting vehicle turns, which are reflected as prominent peaks in this axis. A multi-stage processing framework was implemented to accurately assess driving performance during these turns.

First, a peak-detection algorithm is applied to the gyroscope’s angular velocity data to identify significant peaks corresponding to turning maneuvers. This allows us to isolate the angular velocity associated with each turn. Next, we compute the angular acceleration (α, in rad/s^2^) by calculating the time derivative of the angular velocity data. To ensure precision, a windowing technique is applied around each peak, capturing angular velocity values immediately before and after the turn. The angular acceleration is then determined using the discrete derivative formula:

α = (dω/dt) ≈ (Δω/Δt)(1)

This method enables capturing both the maximum and minimum angular acceleration for each turn, providing a detailed examination of the dynamics during the vehicle’s maneuvers.

In addition to gyroscope data, the GPS speed (m/s) for each turn is also analyzed. Since the GPS data are sampled at 1 Hz and the gyroscope data at 10 Hz, interpolation was used to synchronize the two data streams. This synchronization is crucial for aligning each angular velocity peak with the corresponding GPS speed, allowing us to draw meaningful correlations between turning dynamics and vehicle speed. Furthermore, minor turns, such as smaller adjustments within U-turns, are excluded from the analysis to reduce noise and focus on significant driving performances. This selective focus ensures that only impactful data are incorporated into the final analysis. By concentrating on relevant turns and eliminating extraneous movements, this methodology provides a highly detailed and nuanced assessment of driving performance.

**Figure 4:**
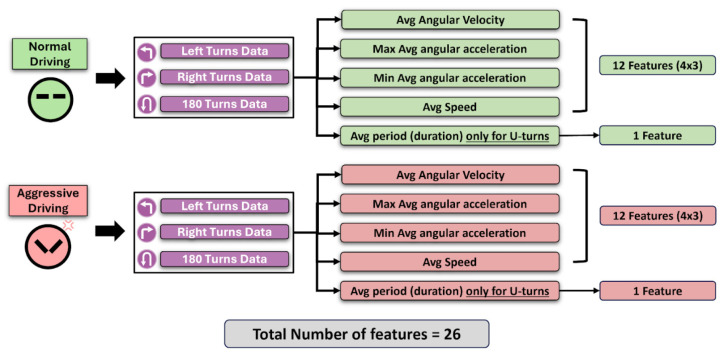
Summary of the final 26 features included in the study’s standardized test. The features were extracted for turning maneuvers during left turns, right turns, and U-turns for both normal and aggressive driving conditions. Summarized statistics such as average (Avg), maximum (Max), and minimum (Min) values were used to represent the sensor readings.

Once these values are extracted and processed, the mean values of the sensor data during the turns are used as features. Figure 4 and Table 2 summarize the features included in the tests.

### 2.4. Machine Learning Models Training

**Figure 5:**
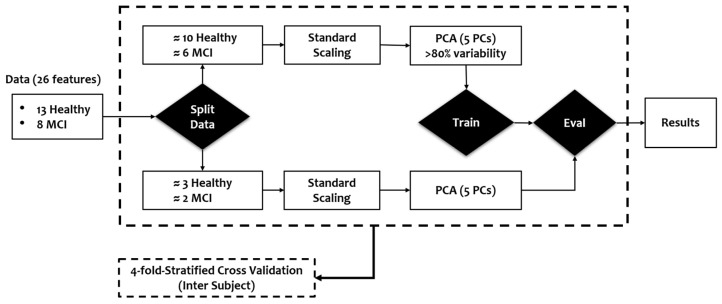
Machine learning modeling flowchart: The dataset is divided using a 4-fold inter-subject cross-validation approach, with each fold allocating around 16 subjects (10 healthy, 6 MCI) for training and 5 subjects (3 healthy, 2 MCI) for validation.

After extracting features from the turning maneuvers data, the features were used to build machine learning models for detecting mild cognitive impairment (MCI). Unlike previous studies, where data from the same subjects could appear in both training and validation sets (potentially inflating performance metrics), our approach uses a 4-fold inter-subject cross-validation. In this setup, the dataset is divided into four groups (or ’folds’), and each fold is used as the validation set, while the remaining three folds are used for training. Importantly, inter-subject cross-validation ensures that data from a single subject are only included in one-fold, preventing any overlap between training and validation sets. Although this approach makes the prediction task harder for the model, it does yield a more realistic and reliable assessment of the model’s ability to generalize to unseen subjects, enhancing its potential for real-world applications. Figure 5 provides a flowchart of the experiment design, wherein, in each fold, 15–16 subjects are used for training and 5–6 subjects for validation, as detailed in Table 3.

**Figure 6:**
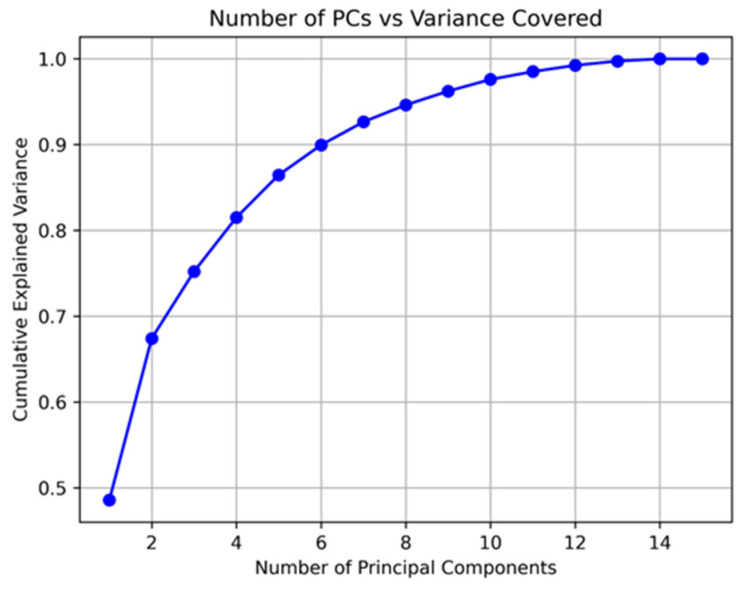
Cumulative explained variance vs. number of principal components. We retained 5 PCs, which accounted for approximately 85% of the total variance.

The collected features were scaled to standardize the data. Subsequently, Principal Component Analysis (PCA) was applied for automatic feature extraction. We retained 5 principal components (PCs), which accounted for approximately 85% of the total variance. Figure 6 illustrates the amount of variability covered by the number of principal components used.

The transformed features were then used to train the machine learning models, and the trained models were evaluated using the validation set for each fold. The final average accuracy across all folds and their standard deviations were then reported.

Six machine-learning algorithms were employed to train and test the models. Table 4 provides a summary of the algorithms along with their standard parameter settings from the Scikit-learn library [26]. The performance of each model was evaluated using the cross-validation framework, allowing us to assess the effectiveness of the algorithms in distinguishing between healthy controls and individuals with MCI.

### 2.5. Evaluation Metrics

To assess the performance of the machine learning models, we calculated several key classification metrics across all folds of the cross-validation process using k = 4 folds. These metrics provide insight into different aspects of model performance, including its overall accuracy, its ability to identify true positives and negatives, and its reliability when making predictions. Table 5 summarizes the metrics used and their mathematical formulation. Each metric was computed for each fold during the cross-validation and then averaged across all folds to obtain the mean value, with the standard deviation (Std) providing a measure of variability across folds.

## 3. Results and Analysis

### 3.1. Classification Results

The algorithms included in this comparative study were Support Vector Machine (SVM), Random Forest, AdaBoost, k-Nearest Neighbors (KNNs), Quadratic Discriminant Analysis (QDA), and Logistic Regression (LR). Table 6 provides a detailed breakdown of the average values for accuracy, sensitivity, specificity, PPV, and NPV, along with their respective standard deviations across all folds for each classifier in identifying healthy controls and individuals with MCI.

**Figure 7:**
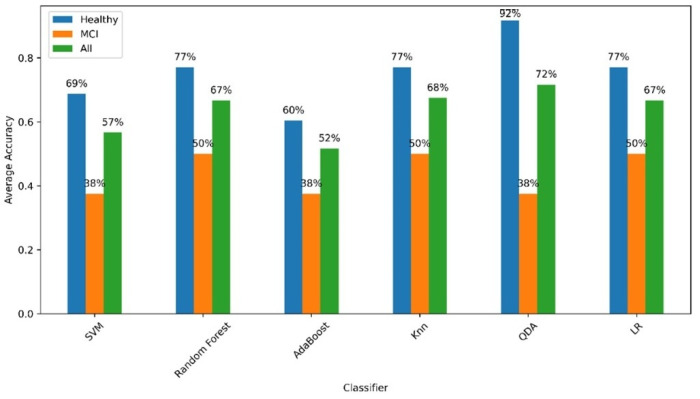
Accuracy of the ML models in diagnosing healthy and MCI subjects. Blue bars represent accuracy for identifying healthy subjects, orange bars indicate accuracy for identifying MCI subjects, and green bars show the overall accuracy across both groups (‘All’).

To further understand the performance of the models, Figure 7 provides a detailed breakdown of the accuracy of each algorithm in identifying healthy controls and individuals with MCI.

As evident from the results, this pilot study highlights the potential of using driving features to classify healthy controls and individuals with MCI while also identifying areas for improvement. Among the models tested, QDA achieved the highest overall mean accuracy (72 ± 10%) and demonstrated strong specificity (92 ± 17%), effectively identifying healthy controls. However, its sensitivity (38 ± 48%) and PPV (42 ± 50%) indicate challenges in reliably detecting MCI. This pattern of high specificity but lower sensitivity across models reflects the complexity of distinguishing subtle cognitive impairments, particularly in a pilot study with a limited dataset.

KNNs (68 ± 15%) and Random Forest (67 ± 19%) demonstrated more balanced performance, with improved sensitivity (50%) and specificity (77 ± 16% and 77 ± 31%, respectively). KNNs achieved higher PPV (54 ± 42%) and NPV (75 ± 18%), while Random Forest exhibited slightly more consistent results, with PPV (71 ± 34%) and NPV (69 ± 12%). Both models show promise for further development. Logistic Regression (67 ± 9%) also performed comparably, with balanced sensitivity (50%) and specificity (77 ± 16%) and relatively stable results across folds, making it another candidate for refinement.

In contrast, SVM (57 ± 12%) and AdaBoost (52 ± 14%) exhibited lower overall accuracy and struggled to achieve satisfactory sensitivity (38 ± 25% for both) and specificity (SVM: 69 ± 28%, AdaBoost: 60 ± 18%). Their PPV (SVM: 33 ± 24%, AdaBoost: 33 ± 24%) and NPV (SVM: 63 ± 11%, AdaBoost: 63 ± 12%) suggest limited effectiveness for MCI detection in this dataset, indicating that further optimization is necessary for these models.

In summary, QDA shows potential in identifying healthy controls, while KNNs, Random Forest, and Logistic Regression demonstrate more balanced performance across the two classes. These findings highlight the feasibility of using machine learning for MCI detection, even at this early stage. However, the results also underscore the preliminary nature of this pilot study, particularly given the small dataset. Future work will focus on refining these models and expanding the dataset to improve sensitivity and overall reliability, especially for identifying MCI cases.

It is worth mentioning that in the process of defining the diagnostic accuracy of the machine learning models to discriminate healthy from MCI older adults, we used exclusively driving performance parameters without any association with the driver’s demographic information (age/sex/ethnicity/education), which has been reported to improved accuracies from 66% to 88% [20], and 82% to 88% [19]. This emphasizes the capability of these features in predicting the MCI status of individuals. In this context, QDA demonstrated superior performance in overall classification accuracy, particularly in identifying healthy controls. KNNs, LR, and Random Forest also showed promising results with varying degrees of accuracy for healthy and MCI subjects. In contrast, SVM and AdaBoost were less effective, indicating a need for further optimization or alternative approaches to enhance the model’s ability to distinguish MCI from healthy controls.

### 3.2. Analysis of Original Features Contribution

To further understand the contribution of the original features to the accuracy of the diagnosis as a healthy or MCI, we analyzed the loadings of each parameter on each of the five PCs used for training. The loadings provide insights into the contribution of each original parameter to the principal components, revealing which features were the most influential to a specific PC. To understand the overall contribution of a specific parameter (not just a single PC), we derived a metric that was calculated as the product of the absolute loadings of each feature per principal component and the variance explained by that specific principal component. These contributions were summed across all the top five principal components for each fold. The equations below walk through the calculations made to obtain the overall feature contribution metric.

Let the elements of vector *γ* represent the variance explained by each PC (*i* = 1, … 5). And let the elements of the |*W*| Matrix represent the absolute value of the loadings of feature *j* in PC *i*.

(2) $$\gamma =\left[\begin{array}{c} \gamma_{1}\\ \vdots \\ \gamma_{5} \end{array}\right],W=\left[\begin{array}{ccc} w_{1,1} & \cdots & w_{26,1}\\ \vdots & \ddots & \vdots \\ w_{1,5} & \cdots & w_{26,5} \end{array}\right]$$

We multiply the loading of each feature by the variance explained by each principal component (PC). This approach provides a more realistic assessment of each feature’s influence, as it accounts for the fact that not all PCs contribute equally to the total variance. By broadcasting the explained variance vector γ and applying element-wise multiplication, we obtain the following matrix:

(3) $$\left|W\right|⊙\gamma =\left[\begin{array}{ccc} \left|w_{1,1}\right| & \cdots & \left|w_{26,1}\right|\\ \vdots & \ddots & \vdots \\ \left|w_{1,5}\right| & \cdots & \left|w_{26,5}\right| \end{array}\right]⊙\left[\begin{array}{ccc} \gamma_{1} & \cdots & \gamma_{1}\\ \vdots & \cdots & \vdots \\ \gamma_{5} & \cdots & \gamma_{5} \end{array}\right]=\left[\begin{array}{ccc} \left|w_{1,1}\right|\cdot \gamma_{1} & \cdots & \left|w_{26,1}\right|\cdot \gamma_{1}\\ \vdots & \ddots & \vdots \\ \left|w_{1,5}\right|\cdot \gamma_{5} & \cdots & \left|w_{26,5}\right|\cdot \gamma_{5} \end{array}\right]$$

Finally, we derive a measure of the individual feature contribution across all principal components by summing over all the columns as such:

(4) $${FeatureContribution}_{j}=\sum_{i=1}^{5}\left|w_{i,j}\right|\cdot \gamma_{i}$$

This generalized form shows how the contributions are computed by multiplying the absolute loadings of each parameter by the explained variance in the corresponding principal component and then summing these contributions across all principal components. Finally, the contributions were summed across all folds and sorted from highest to lowest.

**Figure 8:**
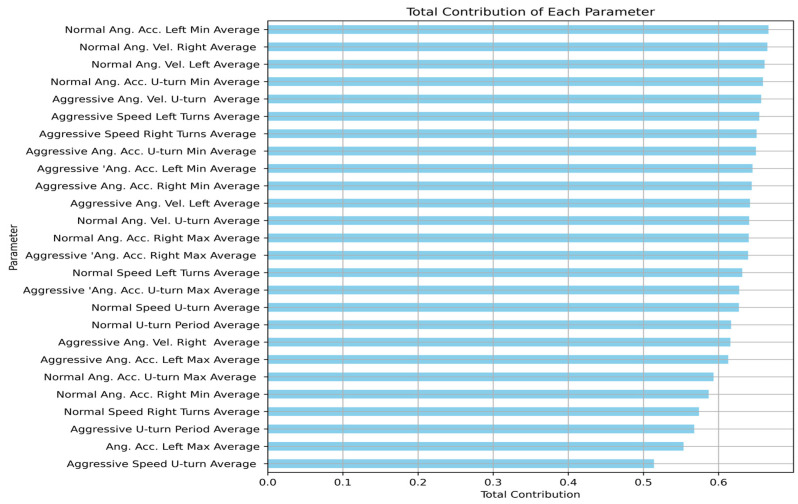
Visualization of the features contributions showcasing the most influential parameters. A higher number signifies a greater contribution to the overall model. Notably, it encompasses both normal and aggressive driving performance parameters, highlighting a comprehensive approach to understanding driving patterns.

Figure 8 shows a visualization of the feature’s contributions in a bar chart to identify the most influential parameters, where a higher number signifies a greater contribution to the overall model. Notably, it encompasses both normal and aggressive driving performances, highlighting a comprehensive approach to understanding driving patterns. Features related to angular acceleration and velocity during various maneuvers emerge as significant contributors, indicating the crucial role of these factors in parameter identification. Moreover, features associated with the speed and duration during U-turns showed fewer overall contributions. While certain features exhibit slightly higher contribution scores, the multidimensional nature of driving performance underscores the significance of each parameter in the overall analysis. These findings have implications for smart driving systems, performance modeling, and MCI evaluations, offering insights for targeted interventions and strategies to improve MCI detection in patients. Future research could explore the interplay between these influential parameters and their impact on MCI detection.

## 4. Discussion

The findings of this pilot study demonstrate the potential of driving performance data as a novel means for detecting MCI. While the results are promising, they also highlight the need for further research and refinement to enhance the model’s performance, particularly in identifying MCI. Below, we provide an expanded discussion of the implications, limitations, and future directions for this pilot study.

### 4.1. LiveDrive AI Implications

This study serves as a foundational step toward integrating driving performance analysis into the toolkit for early cognitive health monitoring. The ability to collect driving data passively and non-invasively offers distinct advantages over traditional diagnostic methods such as the Montreal Cognitive Assessment (MoCA). While MoCA requires individuals to actively seek testing and attend clinical appointments, it is inherently limited as a single-time assessment that does not account for the dynamic and evolving nature of the human brain influenced by time and environmental factors. In contrast, our proposed approach enables continuous monitoring during routine activities, capturing subtle, time-dependent changes in cognitive health that might otherwise go unnoticed. This capability not only reflects the complex nature of cognitive decline but also has the potential to identify early indicators of MCI, encouraging individuals who might delay or avoid clinical testing to seek medical evaluation earlier.

Additionally, the use of everyday driving performance data aligns with the broader goal of developing practical and scalable solutions for cognitive health monitoring. Many modern vehicles are already equipped with sensors capable of capturing the necessary data, and even older vehicles can be supplemented with smartphone applications. This flexibility enhances the feasibility and accessibility of implementing such a system on a large scale. Moreover, integrating this technology into vehicle operating systems as an application could provide real-time feedback and recommendations, making it a valuable tool for both individuals and healthcare providers.

We believe this approach is not only specific to cognitive health monitoring but also represents a generic framework for anomaly detection in driving performance. Depending on the scope of the study, the system could be adapted for various purposes, such as detecting driving under the influence (DUI), where the anomaly would be impaired driving performance instead of cognitive decline. This adaptability highlights the versatility and scalability of the proposed system, making it applicable to a wide range of domains where performance anomalies are indicative of potential risks.

By leveraging continuous data collection and non-invasive monitoring, this approach has the potential to complement traditional methods like MoCA, providing a more comprehensive and proactive strategy for early detection and intervention. Furthermore, its broader applicability reinforces its value as a versatile and innovative tool for addressing various challenges in driving safety and health monitoring.

### 4.2. Study Limitations

Despite its potential, this study’s findings also underscore several limitations that warrant further attention and refinement. One notable limitation is the relatively low sensitivity of the models in detecting MCI, which reflects the inherent challenges of identifying subtle cognitive changes in the early stages of the condition. MCI often presents nuanced and variable symptoms, making it difficult for machine learning models to reliably differentiate it from healthy cognitive functioning. This limitation highlights the need for further advancements in feature extraction and model optimization to enhance sensitivity while maintaining specificity.

Another important consideration that we suspect heavily affected the performance is the small sample size used in this pilot study, which constrains the generalizability of the results. This challenge is particularly relevant in research focused on detecting early cognitive decline, such as MCI, where the recruitment and accurate classification of participants pose substantial difficulties. This study was designed to monitor driving performance among older adults aged 65 to 85, with a primary goal of identifying early signs of cognitive decline. Despite thorough recruitment efforts, only 21 participants were enrolled, comprising 13 cognitively healthy individuals and 8 diagnosed with MCI based on evaluations by a neurologist specializing in AD. The recruitment process necessitated collaboration with medical experts to ensure accurate diagnosis and group classification. This careful but resource-intensive process limited the number of participants that could be included. Additionally, identifying eligible subjects for such studies is inherently challenging due to several factors. First, older adults who may be at risk for cognitive decline are often reluctant to participate in research that might reveal potential health concerns. Second, the diagnosis of MCI itself requires a nuanced understanding of clinical symptoms, often involving detailed neuropsychological assessments, which further narrows the pool of eligible participants. Lastly, ensuring informed consent and providing adequate education about this study’s goals and methods add additional layers of complexity to the recruitment process. These factors, while necessary for scientific rigor, underscore the inherent difficulty in obtaining a sufficiently large and diverse sample. A larger dataset would enable more robust statistical analyses and improve the models’ ability to generalize across broader populations. Future research should focus on scaling up participant recruitment through multi-site collaborations, leveraging larger research networks, and employing innovative strategies such as remote data collection to increase accessibility for potential participants.

Finally, this study’s focus on driving performance data exclusively, while intentional, represents another limitation. This approach was designed to demonstrate the standalone predictive utility of driving patterns, emphasizing their potential as non-invasive indicators of cognitive health. However, it also meant excluding other potentially valuable features such as demographic variables, medical history, or multimodal biomarkers (e.g., genetic or neuropsychological data). Future research could benefit from integrating these complementary features; this could enhance the models’ predictive power and provide a more holistic understanding of cognitive health.

Despite these limitations, this study’s pilot nature provides valuable preliminary insights. Importantly, it demonstrates that driving behavior data alone holds promise as a non-invasive indicator of cognitive health. By addressing the recruitment challenges and expanding the sample size in future studies, the potential of this approach can be further validated and refined, paving the way for more impactful applications in early MCI detection. In the next subsection, suggested future directions are discussed to build upon this pilot study and overcome these limitations.

### 4.3. Future Directions

Addressing the limitations of this study and advancing the field of cognitive health monitoring through driving performance analysis will require several key strategies. First, expanding the sample size is essential for enhancing the reliability and generalizability of the models. A larger dataset would not only improve model robustness but also enable more detailed analyses across different demographic and clinical subgroups, providing insights into how performance varies among diverse populations. By including a broader range of participants, future studies can better evaluate the applicability of these methods in real-world settings.

Incorporating additional novel features into the models offers another promising avenue for improvement. One possible example is the addition of features such as metabolism, which could be an indicator of how much oxygen the brain consumes while driving. Another direction could be longitudinal data capturing changes in driving performance over time, geospatial features that account for environmental and contextual factors, and multimodal biomarkers that could significantly enhance predictive accuracy. These additional data sources would provide a richer, more nuanced understanding of cognitive health and allow for a more comprehensive approach to early detection. Geospatial data, for instance, could reveal driving patterns influenced by route complexity or familiarity, offering valuable context for interpreting behavioral anomalies.

Advanced modeling techniques will also play a critical role in future research. Deep learning models specifically designed for time-series data, such as recurrent neural networks (RNNs) or transformer-based architectures, could more effectively capture complex temporal dynamics in driving performance. These models, combined with innovative feature extraction methods, could unlock hidden patterns in geospatial and behavioral data that are not immediately apparent through traditional approaches. Smart feature extraction, for example, could identify driving anomalies related to navigation errors or reaction times in challenging traffic scenarios, further differentiating individuals with MCI from healthy controls. However, it should be noted that deep learning models are data-intensive, and their performance could be hindered by the current sample size.

Longitudinal studies represent a particularly important step forward. By tracking participants’ driving behaviors over extended periods, researchers can observe subtle changes that may serve as early indicators of cognitive decline. This approach would allow for the identification of precursors to MCI before clinical symptoms manifest, providing critical opportunities for early intervention. Longitudinal monitoring could also help refine models by revealing how cognitive decline progresses over time, supporting more personalized and adaptive strategies for intervention.

By addressing these future directions, this line of research can contribute to a deeper understanding of cognitive health and support the development of innovative, practical tools for early detection and intervention.

## 5. Conclusions

In conclusion, this work introduces LiveDrive AI, a machine learning-powered diagnostic system for non-invasive, real-time detection of cognitive decline associated with Alzheimer’s disease and related dementia. The system integrates a standardized driving test designed to capture driving performance under varied stress conditions, such as normal and aggressive driving, enabling a more nuanced assessment of cognitive engagement. Additionally, LiveDrive AI employs smart feature extraction techniques focused on turning data, such as angular acceleration and velocity, to reveal subtle indicators of cognitive health through specific driving maneuvers. Together, these components contribute to a comprehensive framework for evaluating real-world cognitive function in older adults. Leveraging expertly annotated test subjects to capture complex driving performance features as digital biomarkers, LiveDrive AI enables clinically informed analysis of cognitive health. Notably, the system addresses inter-subject variability, enhancing the robustness and generalizability of machine learning models in detecting MCI and ensuring the reporting of non-inflated results. The results of the evaluation demonstrate that QDA achieved the highest overall accuracy (72%), with effectiveness in identifying healthy individuals, though MCI classification still presents areas for improvement. Furthermore, the analysis of feature contributions revealed that parameters related to angular acceleration and velocity during various maneuvers are significant for model performance, while features associated with speed and duration during U-turns contributed less. These insights underscore the complexity and importance of multiple driving performance parameters in MCI detection.

This pilot study presented in this work serves as a foundational step toward transitioning from controlled driving settings to real-world, naturalistic environments where participants’ everyday driving patterns can be observed. Building on these initial insights, the next phase will focus on leveraging advanced methodologies to refine and expand the scope of analysis.

In future research, deep learning models designed for time series data, such as RNNs or transformers, could play a pivotal role in capturing temporal dependencies in naturalistic driving data. These models are particularly suited for processing sequential data like driving patterns, enabling the identification of subtle behavioral shifts associated with MCI. While also considering the need to optimize models through hyperparameter tuning and ensemble methods to better balance sensitivity and specificity. Moreover, incorporating smart feature extraction techniques from geospatial data may provide richer contextual insights into driving performance and its relationship with cognitive health. Furthermore, expanding the dataset to include larger and more diverse populations will enhance the generalizability of these approaches. The integration of multimodal data, including biometric modalities such as the ones obtained using wearable sensor data, could further strengthen the predictive capabilities of the models. These advancements aim to guide the development of a more comprehensive LiveDrive AI system capable of continuous, non-invasive monitoring of cognitive health in everyday settings. Ultimately, this transition to naturalistic driving analysis will deepen our understanding of real-world performance, improve early detection of MCI, and support personalized intervention strategies tailored to individual needs.

## Figures and Tables

**Table 1:** Participant demographics and group classification: The study included 21 elderly participants aged 65 to 85, with a gender distribution of 11 males and 10 females. Based on evaluations by medical experts, participants were divided into two groups: 13 were identified as healthy, while 8 were classified as having mild cognitive impairment (MCI).

Subject No.	Age	Sex	Status
Subject 1	78	Male	MCI
Subject 2	80	Male	Healthy
Subject 3	70	Male	Healthy
Subject 4	73	Female	Healthy
Subject 5	74	Male	MCI
Subject 6	77	Female	Healthy
Subject 7	87	Female	Healthy
Subject 8	85	Female	Healthy
Subject 9	82	Female	MCI
Subject 10	71	Female	Healthy
Subject 11	72	Female	MCI
Subject 12	65	Male	MCI
Subject 13	75	Male	Healthy
Subject 14	71	Female	MCI
Subject 15	76	Male	Healthy
Subject 16	75	Female	MCI
Subject 17	83	Male	Healthy
Subject 18	76	Male	MCI
Subject 19	80	Female	Healthy
Subject 20	79	Male	Healthy
Subject 21	75	Male	Healthy

**Table 2:** Summary of the features included in standardized tests. A total of 26 features were captured, representing the mean values of the sensor data. These features included measures of angular velocity, angular acceleration, speed during left and right turns, U-turns, and the period of U-turns. The features were categorized into normal and aggressive driving styles.

Feature	Feature Description	Unit
1	Average angular velocity during normal left turns	rad/s
2	Average angular velocity during normal right turns	rad/s
3	Average angular velocity during normal U-turns	rad/s
4	Average angular velocity during aggressive left turns	rad/s
5	Average angular velocity during aggressive right turns	rad/s
6	Average angular velocity during aggressive U-turns	rad/s
7	Maximum average angular acceleration during normal left turns	rad/s^2^
8	Minimum average angular acceleration during normal left turns	rad/s^2^
9	Maximum average angular acceleration during normal right turns	rad/s^2^
10	Minimum average angular acceleration during normal right turns	rad/s^2^
11	Maximum average angular acceleration during normal U-turns	rad/s^2^
12	Minimum average angular acceleration during normal U-turns	rad/s^2^
13	Maximum average angular acceleration during aggressive left turns	rad/s^2^
14	Minimum average angular acceleration during aggressive left turns	rad/s^2^
15	Maximum average angular acceleration during aggressive right turns	rad/s^2^
16	Minimum average angular acceleration during aggressive right turns	rad/s^2^
17	Maximum average angular acceleration during aggressive U-turns	rad/s^2^
18	Minimum average angular acceleration during aggressive U-turns	rad/s^2^
19	Average speed during normal left turns	m/s
20	Average speed during normal right turns	m/s
21	Average speed during normal U-turns	m/s
22	Average speed during aggressive left turns	m/s
23	Average speed during aggressive right turns	m/s
24	Average speed during aggressive U-turns	m/s
25	Average period (duration) of normal U-turns	s
26	Average period (duration) of aggressive U-turns	s

**Table 3:** IDs and counts of MCI and healthy subjects in training and testing sets across cross-validation folds.

Fold	Training Data Subjects		Testing Data Subjects	
	MCI	Healthy Subjects	MCI	Healthy Subjects
	Subjects ID (N = Count)	Subjects ID (N = Count)	Subjects ID (N = Count)	Subjects ID (N = Count)
1	6, 12, 13, 15, 17, 19 (N = 6)	3, 5, 8, 9, 14, 16, 18, 21, 22 (N = 9)	1, 10 (N = 2)	2, 7, 11, 20 (N = 4)
2	1, 6, 10, 12, 15, 19 (N = 6)	2, 5, 7, 8, 9, 11, 16, 18, 20, 21 (N = 10)	13, 17 (N = 2)	3, 14, 22 (N = 3)
3	1, 10, 12, 13, 17, 19 (N = 6)	2, 3, 5, 7, 9, 11, 14, 18, 20, 22 (N = 10)	6, 15 (N = 2)	8, 16, 21 (N = 3)
4	1, 6, 10, 13, 15, 17 (N = 6)	2, 3, 7, 8, 11, 14, 16, 20, 21, 22 (N = 10)	12, 19 (N = 2)	5, 9, 18 (N = 3)

**Table 4:** Summary of the machine learning models used in this experiment, along with the selected hyperparameters for each model. For definitions and descriptions of these hyperparameters, please refer to the scikit-learn documentation of the specific ML model (https://scikit-learn.org/stable/supervised_learning.html, accessed on 12 January 2025.).

Model Name	Model Parameters Settings
Support Vector Machine (SVM)	C = 1.0, kernel = ‘rbf’, gamma = ‘scale’
Random Forest	n_estimators = 100, criterion = ‘gini’, max_depth = None, min_samples_split = 2, min_samples_leaf = 1
AdaBoost	n_estimators = 50, learning_rate = 1.0
k-Nearest Neighbors (KNN)	n_neighbors = 5, algorithm = ‘auto’, metric = ‘minkowski’, p = 2
Quadratic Discriminant Analysis (QDA)	priors = None, reg_param = 0.0
Logistic Regression	penalty = ‘l2’, C = 1.0, solver = ‘lbfgs’, max_iter = 100

**Table 5:** Overview of the evaluation metrics and their mathematical formulations, including accuracy, sensitivity, specificity, positive predictive value (PPV), and negative predictive value (NPV). Abbreviations: TP (True Positive), FP (False Positive), TN (True Negative), FN (False Negative).

Metric	Equation
Accuracy	$\frac{TP+TN}{TP+TN+FP+FN}$
Sensitivity (Recall)	$\frac{TP}{TP+FN}$
Specificity	$\frac{TN}{TN+FP}$
Positive Predictive Value (PPV)	$\frac{TP}{TP+FP}$
Negative Predictive Value (NPV)	$\frac{TN}{TN+FN}$

**Table 6:** Summary of the classification performance of the machine learning models across all folds. The table presents the average values of accuracy, sensitivity, specificity, PPV, and NPV, along with their respective standard deviations across all folds.

Model	Accuracy (Mean ± Std)	Sensitivity (Mean ± Std)	Specificity (Mean ± Std)	PPV (Mean ± Std)	NPV (Mean ± Std)
SVM	57 ± 12%	38 ± 25%	69 ± 28%	33 ± 24%	63 ± 11%
Random Forest	67 ± 19%	50 ± 0%	77 ± 31%	71 ± 34%	69 ± 12%
AdaBoost	52 ± 14%	38 ± 25%	60 ± 18%	33 ± 24%	60 ± 13%
KNNs	68 ± 15%	50 ± 41%	77 ± 16%	54 ± 42%	75 ± 18%
QDA	72 ± 10%	38 ± 48%	92 ± 17%	42 ± 50%	75 ± 18%
LR	67 ± 9%	50 ± 0%	77 ± 16%	62 ± 25%	71 ± 5%

## Data Availability

The datasets presented in this article are not readily available because the data are part of an ongoing study. Requests to access the datasets should be directed to Erica Forzani (eforzani@asu.edu).
